# Ethical reflections of healthcare staff on ‘consentless measures’ in somatic care: A qualitative study

**DOI:** 10.1177/09697330251328649

**Published:** 2025-04-14

**Authors:** Joar Björk, Niklas Juth, Tove Godskesen

**Affiliations:** Centre for Research Ethics & Bioethics, 8097 Uppsala University; Swedish National Centre for Priorities in Health, Linköping University; Centre for Research Ethics & Bioethics, 8097 Uppsala University; Centre for Healthcare Ethics, Karolinska Institutet; Centre for Research Ethics & Bioethics, Uppsala University; Faculty of Nursing and Health Sciences, 8097 Nord University

**Keywords:** Attitudes, autonomy, clinical ethics, coercion, decision-making capacity, healthcare staff, informed consent, nurses

## Abstract

**Background:**

Many patients in medical wards lack decision-making capacity and cannot provide valid consent. As a result, nurses and other healthcare professionals often face a dilemma: whether to neglect the medical needs of such patients, or provide healthcare interventions without obtaining valid consent. Previous studies have indicated that many interventions are provided without consent; however, there is insufficient knowledge about how staff in this context reason about the ethical dilemmas they encounter.

**Aim:**

To explore the ethical reasons provided by nurses and other healthcare professionals in medical wards for and against providing healthcare interventions without patients’ consent.

**Research design:**

The study employed a qualitative explorative design. Eight focus group interviews were held with 37 staff across five different professions, mainly nurses, at two Swedish hospitals. The material was subjected to qualitative analysis, following a Reflective Thematic Analysis framework.

**Ethical considerations:**

Ethical approval for this study was obtained from the Swedish Ethical Review Authority. All participants were informed orally and in writing about the study’s aims and its voluntary nature. No sensitive personal information was registered. Participants provided their oral consent to participate before the interviews took place.

**Findings/Results:**

Thematic analysis resulted in four main themes: *Coercion is a bad word*; *Reasons to accept coercion*; *Coercion is part of ward culture,* and *Unacceptable coercion.*

**Conclusions:**

Participants overwhelmingly supported the current use of ‘consentless measures’ at the investigated wards. Most situations described either needed no justification, according to participants, or could be easily justified by reference to the benefit of the patient, the patient’s poor decision-making capacity, or the benefit of others. A range of implicit, contextual, and institutional justifications were also given. Suboptimal ward culture was considered a prime driver of consentless measures and a force that compromises nurses’ agency in the patient encounter.

## Introduction

This qualitative study explores how nurses and other healthcare staff in Swedish medical wards justify providing medical interventions in the absence of patients’ consent. The findings indicate that such interventions are common, and that staff generally find them easy to justify. Although ethical reflections about interventions without consent have been much studied within psychiatry, there has been few previous studies from the somatic (medical) context. This study adds to the understanding of staffs’ ethical reasoning on this important topic.

## Background

Ideally, healthcare should be guided by patients’ informed consent: the healthcare provider proposes an intervention based upon the patient’s health condition, and the patient provides valid consent.^1^ However, healthcare is sometimes delivered without valid consent, for example when the patient lacks the necessary capacity to consent. Coercive psychiatric care is a paradigmatic example^2^: treatments and other interventions are administrated without the patient´s consent but with legal authorisation. In somatic care, the regulatory scaffolding is often less clearly articulated, though previous studies suggest that treatment without consent is nonetheless common.^3–5^ Although all health professionals may encounter coercion-related dilemmas, nurses are particularly exposed due to their proximity to patients.^6,7^

Patients’ consent is a complex topic. There is no consensus on which interventions require informed consent or which criteria must be met for consent to be considered ‘informed’.^8,9^ A related issue in inpatient care involves whether consent to, for example, a blood test on 1 day implies consent to another test on a subsequent day. The use of presumed or blanket consent can blur the boundaries of what patients have agreed to.^10^ Moreover, attempts to secure patients’ consent by explaining interventions often prioritise legal or practical aspects over the ethical values inherent to consent.^11,12^ Additionally, healthcare staff (henceforth: staff) may overestimate patients’ decision-making capacity and health literacy, leading to misjudgements about their ability to provide valid consent.^13^ This concern applies to nurses as much as to other staff.^14^ Empirical studies suggest that a significant proportion of patients in medical wards, similar to those in psychiatric hospitals, actually lack the capacity for informed consent.^10,15^

This raises complex ethical questions about the justification of healthcare and the safeguarding of patients’ integrity and broader well-being interests. Nurses and other staff play a central role in these challenges. However, there is limited knowledge about the experiences and perspectives of staff on coercive and borderline coercive measures in somatic healthcare—particularly regarding ethical considerations.^16^ Tentative insights indicate that staff sometimes resort to coercive measures to ensure medical treatment, maintain ward safety, and secure decent working conditions.^17–19^ Staff report feeling caught in an ethical ‘grey zone’, often without adequate guidance on appropriate courses of action.^17,20,21^

## Aim

The current study aimed to contribute to a deeper understanding of how staff in medical wards reason about the ethical and practical dimensions of providing interventions without patient consent.

## Method

For a full report of our method according to the COREQ template, see Appendix 1.

### Research design

This study employed a qualitative explorative design.

### Context and recruitment

The study was conducted in medical wards at two Swedish hospitals: one with a total of 400 beds and one in a university hospital with 1000 beds. Medical wards were chosen because they cater to a diverse patient population, including elderly patients with significant medical and care needs. Swedish law permits no coercive somatic treatment outside of acute emergencies,^22^ so any coercive measures were expected to be informal. Furthermore, Sweden stands out internationally by affording a minimal role in medical decision-making to relatives of patients with impaired decision-making capacity.^23,24^

Participants were recruited purposefully through ward operation managers, with the aim of capturing variation in work experience (minimum 1 year). Since coercion may be initiated by or affect various healthcare professions, diversity in professional backgrounds was also sought. Potential participants were informed about the voluntary nature of the study both in writing and verbally and were guaranteed anonymity. Eight focus group interviews were conducted with 37 participants, mostly nurses. None of the potential participants declined to participate. For demographic details, see Table 1.

**Table 1.** Participant demographics.

Number of focus group interviews	8
Total number of participants (participants per focus group interview)	37 (4–6)
Professions represented	
Registered nurses (RN)	13
Assistant nurses (AN)	15
Medical doctors (MD)	5
Physiotherapists (PT)	2
Occupational therapists (OT)	2
Sex distribution (female/male)	33/4
Age, mean (range)	38 (22–64)
Years worked on a medical ward, mean (range)	12 (1–48)

### Data collection

Three individual pilot interviews were conducted. The main takeaway was that focussing explicitly on ‘coercion’ hindered the discussion, as many ethically problematic actions on medical wards were not perceived as coercive but as existing in a grey zone just beyond clear, irrefutable coercion. This insight informed the interview guide, which was subsequently pilot tested with good results. The semi-structured interview guide began with a broad definition of ‘consentless measures’ to capture the grey zone identified during pilot testing. For the full interview guide, see Table 2. Results of pilot testing are not reported.

**Table 2.** Interview guide.

In this interview, the term ‘consentless measures’ will be used broadly for actions to make patients comply with treatment - examples include actions such as coaxing, deception, non-voluntary medication, and use of physical force
Are consentless measures, in this sense, used at this ward?
What kind of patients are subjected to consentless measures?
Can you describe some measures frequently used?
How do staff learn to use and master these measures?
Please share your thoughts and reflections about the usage of consentless measures?
Are situations involving consentless measures discussed among staff?

Focus group interviews were used to collect data, with the aim of capturing not only individual reflections but also collectively shared attitudes that influence healthcare interventions in the absence of patient consent.^25^ Interviews were conducted by the first author in a private room at the medical department, and ranged from 53 to 81 minutes in length. After seven focus group interviews, the research team concluded that sufficient information had been collected for comprehensive analysis to yield a rich understanding of the topic. To ensure completeness, one additional focus group interview was conducted. Best practices for focus group interviews, such as using open-ended questions and encouraging further reflection, were followed.^25^ Interviews were recorded and transcribed by a specialist company.

### Data analysis

Reflexive Thematic Analysis, as described by Braun and Clark and developed by Byrne, was selected for use in this study to enable an in-depth exploration of participants’ thoughts and reasoning.^26,27^ Data analysis was conducted in six phases: (1) familiarisation with the data; (2) generating codes; (3) developing themes; (4) reviewing themes; (5) refining and defining themes; and (6) producing the final report.

The primary analytical work was conducted by the first author, with the second and third authors reviewing excerpts and making significant contributions to analysis and theme generation. In the initial phase, JB read each interview multiple times to become familiar with the data, noting initial ideas and highlighting striking passages. Next, JB identified patterns and structured content relevant to the study’s focus, generating meaningful codes by systematically labelling significant features of the data.

In the third phase, the codes were organised into themes and sub-themes, grouping related codes to form overarching themes that captured the essence of the data. During the fourth phase, JB led the analysis while all authors refined the themes, reassessing their alignment with the coded data to ensure they were coherent and accurately represented the participants’ narratives. The themes were then reviewed to ensure conceptual distinctiveness and clarity.

The analysis aimed to go beyond the manifest content to capture latent attitudes expressed through participant interactions and clinical examples shared during interviews. Microsoft Excel software was used to organise the analysis. In the final phase, all authors independently reviewed the themes to reach consensus, ensuring that the thematic structure was robust and comprehensive. In the final phase, JB wrote up the findings, presenting a coherent narrative that reflects the study’s insights. All authors provided feedback and revisions, further strengthening the final thematic structure.

### Ethical considerations

Ethical approval for the study was sought from the Swedish Ethical Review Authority (registration number 2022-00142-01). In their response, the authority determined that the study did not require formal approval. Prospective participants were informed both orally and in writing about the nature of the study and the voluntary nature of their participation. No sensitive personal information was collected. Before the focus group interviews, participants provided oral consent for both their participation and the publication of results. Confidentiality was ensured by removing all personal identifiers, and findings are reported in a manner that prevents identification of participants. Interview transcripts and the key code were stored separately from the audio files on two secured computers.

## Findings

The analysis of participants’ reasoning about the ethics and practicalities of providing healthcare interventions without consent yielded four themes: *Coercion is a Bad Word; Reasons to Accept Coercion* (sub-themes: *Implicit Justifications* and *Explicit Justifications*)*; Coercion is Part of Ward Culture;* and *Unacceptable Coercion* (see Table 3). ‘Participants’ refers to individuals in this study, while ‘staff’ denotes all healthcare personnel.

**Table 3.** Themes and sub-themes.

Coercion is a bad word
Reasons to accept coercion
*Implicit justifications*
*Explicit justifications*
Coercion is part of ward culture	
Unacceptable coercion	

### Coercion is a bad word

Participants struggled to find appropriate terms for the consentless measures that occurred daily in their workplaces. Although they occasionally described some situations as ‘coercive’, they generally avoided the term or used softened variations like ‘slightly coercive’ or ‘partially coercive’. The word ‘coercion’ was associated with insensitivity and harshness, which participants emphatically distanced themselves from. One participant, after explaining that most healthcare interventions are administered with patients’ consent, struggled to find a suitable term for interventions carried out without consent:

In some situations when it is really important that the patient [complies], then maybe we use some kind of coercion. Well, perhaps not the kind of coercion you’d think of, like real coercion. But still we would coerce the patient, somehow. (RN, interview B)

Participants also found it difficult to classify coercive actions theoretically. The degree of coercion was instead judged by the context. Participants often used the word ‘feels’ when discussing whether situations were coercive, indicating that emotional factors were present in their assessment. Preferentially used terms tended to be descriptive and specific, such as ‘persuade’, ‘coax’, or ‘deceive’.

### Reasons to accept coercion

Many situations involving consentless measures were described as routine and requiring no justification. In other cases, participants described what they considered to be sufficient justification, which was often implicitly embedded within their narratives. There were also some attempts to discuss general justification of consentless measures.

#### Implicit justifications

The dominant approach described by staff when patients refused interventions was to persuade them to change their minds or proceed with the interventions anyway. Taking refusals at face value was considered unusual and seen as conflicting with essential duties of care. As one nurse remarked, ‘you don’t just give up’. Since ‘giving up’ was viewed as a poor alternative—at least if the intervention was perceived as important—staff often worked hard to manage reluctant patients. Those able to provide healthcare without overt conflict were seen as exemplary, while staff who ‘gave up’ were perceived as uncommitted, sloppy, and unreliable. One nurse illustrated this mindset when discussing how staff dealt with patients who wanted to discontinue ventilator therapy:

We try to push them to keep the treatment (…) We are not very quick to discontinue it. I mean we try to encourage them, that’s not really coercion, but anyway they say they want to discontinue, and we encourage them to continue (…) We check whether we can persuade them like: ‘come on, you can keep it a bit longer’. (RN, Interview A)

According to participants, coaxing and other low-grade coercive measures are frequently used. However, staff prefer to have patients’ consent for all necessary actions. Broad patient consent, which grants staff the freedom to act as they see fit, was described as ideal. Patients who did not readily consent to recommended interventions were described as ‘difficult’, with caring for such patients perceived as ‘tiresome’:

We are here to do good and we want them to get better, but some patients just don’t understand this. Those situations are so tiresome, like you have to work against the patient’s will even though… even though we do the right thing. (RN, interview B)

To navigate the tension between avoiding coercion and not wanting to ‘give up’, staff often started without using coercion, gradually increasing the level of coercion as necessary. Participants generally believed staff applied an appropriate amount of coercion relative to the situation. Mastery of various consentless measures and their skilful application was seen as positive. In the following quote, one participant mocks her own ineffectual strategies to get a patient to the common room, contrasting them with her colleagues’ more draconian strategies:

There were two of them… just like this, straight in under her arms, got her into a wheelchair and drove her off. So, they didn’t talk to her at all… whereas I spent half an hour and didn’t even get her started. For them it was thirty seconds’ work. Not that it’s good, though – they forced her, that’s for sure. (AN, interview B)

Her own evaluation of this event was conflicted. What upset her was not the coercion itself but that her colleagues ‘didn’t talk’ to the patient. Nevertheless, she concluded that their approach was justified because it efficiently achieved the desired outcome. Participants generally found consentless measures to be more justified when performed with a warm and communicative approach. For instance, fitting urinary catheters against patients’ protests was typically seen as objectionable but could be tolerated if good rapport had been established.

#### Explicit justifications

As noted, participants generally offered implicit justifications embedded within narratives about the multifaceted challenges of ward work. However, some explicit, general normative arguments were also presented. The most common justification was that consentless measures were used for patients’ benefit. While some participants emphasised medical benefits, others included broader aspects of well-being. This belief in the beneficial nature of consentless measures sometimes made it difficult for participants to engage in discussions about possible drawbacks:

AN: It’s hard [to assess the ethics of different consentless measures], because at the end of the day, we always do what is best for the patient…

RN: I agree! Sure, sometimes we are a bit rough... But that’s just because we want what’s good and strive for the best for the patient… we never do anything to be mean or to force the patient to do anything, we simply want what’s best. (AN and RN, interview A)

Another argument was that patients do not always understand what is in their best interests. Participants generally found consentless measures easier to justify when patients lacked decision-making capacity, especially if the lack of capacity was directly related to the disease being treated (e.g. neurological diseases or acute infections). However, participants were conflicted about how to proceed when patients with intact capacity refused recommended treatment. Some maintained that patients’ best interests could justify compelling even such patients to comply with treatment, while others disagreed.

At times, participants found it difficult to determine whether a particular action was or had been coercive. They expressed uncertainty about both patients’ preferences and their decision-making capacity:

Is the patient lucid, is the patient demented, is the patient confused, there is just so much

[to consider]? You never really know all those things… (AN, interview C)

Furthermore, when patients change their minds or contradict themselves, it can be difficult to determine whether an intervention conflicts with the patient’s will. Consider this excerpt from a longer passage where a physician described being unsure of whether they had received a valid consent. The physician concluded that the situation was not coercive, as she viewed uncertainty as a justifying factor:

It’s hard to know what is right, really. Because this patient had first said no, and then I coaxed a bit and all of a sudden it was okay. And also, we must do what we have to…well, what we want to do in order to help the patient (MD, interview D)

How patients’ dissent was expressed also influenced participants’ ethical judgements. Violating strongly worded or clearly expressed dissent was considered worse than violating more meekly stated preferences. A nurse reflected on whether it is coercive to provide treatment against the will of a ‘fairly compliant’ patient:

Most are fairly compliant even if they say they don’t want it [treatment]. And that’s… if I think about it from that perspective, I guess that is a kind of coercion, like unconsciously you know the patient has said she doesn’t want it, but still you do it. Even if there is no obvious resistance you still know the patient has said: ‘I don’t want this’. (RN, interview F)

The degree of coercion required was also considered relevant. For example, treatment with antibiotics was generally seen as mild and therefore easier to justify, whereas inserting a urinary catheter required stronger justification. Another factor was related to which measures had already been attempted. Using more coercion was deemed acceptable only if less coercive methods had been tried first. Participants were conflicted about whether nursing interventions, such as enforcing a shower, were more or less coercive than medical treatments. However, actions involving physical force were generally viewed as more problematic than those involving deception or coaxing; as one participant noted: ‘We aren’t allowed to use physical methods. So that’s why we have to use a lot of coaxing’ (AN, interview A).

A final argument justifying consentless measures was that they may be necessary to protect the interests of others. Participants justified forcing one patient to take sedatives if she was disturbing other patients or staff. Similarly, the general workload could justify staff compelling patients to accept certain interventions. In the following passage, a participant explained why staff might insist on placing a urinary catheter:

We see it from both perspectives [the patient’s and our own]. She won’t be able to sleep at all if we have to run in there and change her clothes all the time. But also we see that this uses a tremendous amount of our resources. I mean, we don’t really have time to help her [with changing clothes]. (AN, interview C)

### Coercion is part of ward culture

In addition to justifying consentless measures, participants also described them as deeply ingrained in ward work, to the point where they were seen as beyond good or bad. Some participants had generally positive feelings toward ward culture, while others were more critical. However, all agreed that ward culture emphasises efficiency and tacitly condones the use of consentless measures. The overarching goal is to complete tasks, and patient consent is not a primary concern within the wards’ institutional logic:

You have this goal in front of your eyes, and you go there by all means. On the way you don’t focus on the patient and things like, ‘what does she want, what is her goal?’ So, you get frustrated when the patient doesn’t cooperate because we want to reach the goal, whereas she maybe wants something else. And that’s where, I think, a lot of persuasion and coercion starts. (RN, interview B)

Medical decisions in wards are often made before meeting the patient, by physicians who have limited knowledge of the patient’s wishes. Nurses and assistant nurses, who have more insight into patients’ preferences, were reported to have little influence on decision-making. Additionally, as tasks are delegated within teams, the individual actually administering an intervention may not be aware of the underlying rationale. As a result, staff may be required to provide care that has been decided by somebody else, with little time or opportunity to ensure that the patient is onboard with what is happening.

Furthermore, communication with the patient tends to focus on medical details in a way that discourages the patient’s input. According to participants, patients are rarely asked about their health goals or medical preferences, although such discussions sometimes arise when patients explicitly oppose medical recommendations. Quotes from a physician and an assistant nurse illustrate this:

As a physician I plan, like, ‘this is how I want it and what will be the best’, and I don’t necessarily think so much about what this may mean for the patient (MD, interview D)

It is only after you have pushed on for a long time [in providing care] that you ask the patient what she really wants (AN, interview F)

Participants were aware of a tendency to view older patients differently from younger ones. Older patients are often seen as less capable of managing their own needs, which can lead to a greater inclination to provide healthcare without consent. Participants also noted that older patients are generally more compliant with recommendations, and as a result more susceptible to persuasion. All agreed that older patients are treated more coercively than younger patients in the ward, and many expressed ethical concerns about this. In the following quote, two nurses discuss how ‘people’ (i.e. other staff) act:

RN: I think younger patients know what they want and they will say when they need something or if they don’t want to do something. And then it feels like people accept it more than if an older person had said they did not want to do it.

AN: I agree. People are more accepting of the younger patients. Like if they [younger patients] don’t want to agree to something people don’t force them as much as if it were an older [patient]. There people are more… they just don’t give up. I don’t know why this is so, but I think it is the way it is. (RN and AN, interview G)

### Unacceptable coercion

Participants considered very few of the situations they described to be difficult to justify. Hard-to-justify cases typically involved staff using excessive physical force or performing meaningless interventions. Consider, for example, a case involving perceived excessive physical force:

I had this colleague who held on to [the patient’s] head and forced the medicine into his mouth. And he [the patient] spat and hissed but she really forced it in and I know I had nightmares about that afterward because it was really unpleasant to see. (AN, interview B)

Reflections on potential meaninglessness arose when very ill patients were subjected to interventions with little hope of a positive effect. In such cases, participants found it challenging to justify consentless measures. In the quote below, one participant laments the meaninglessness of a series of interventions that were undertaken despite the patient’s feeble but clear non-consent:

We continue to take blood tests and give fluids, we give everything instead of just… there is nothing indicating that they’ll get better, rather everything just gets worse, they’re dying. But still somehow we don’t want to make that decision so we keep on doing too much. We take tests and prescribe antibiotics and prescribe and prescribe, even though the patient said ‘I don’t want this. I am done here, I don’t want any more’. But still we keep going, and that is just so wrong I think. I am sorry but there it is. (AN, interview C)

## Discussion

This study makes a significant contribution to research on potentially coercive interventions in healthcare by focussing on a less-explored clinical context (medical wards) and employing a deliberately broad framework of discussion (‘consentless measures’). As nurses and other healthcare staff deliberated the ethics and practicalities of consentless measures in somatic care, they reported regularly providing healthcare without consent and found most consentless measures used in their clinical context easy to justify. Core justifications included benefit to patients, patients’ lack of decision-making capacity, and benefit to others. Additionally, contextual features such as good rapport with the patient and careful application of consentless measures to the situation were seen as contributing to justifiability. At the same time, participants reported being part of, or having witnessed, some hard-to-justify practices in this clinical context.

Many previous studies support the finding that staff largely accept consentless measures, perhaps partly due to habituation.^16,17,28–34^ The justifications used by participants in this study are strikingly similar to previous studies even though the context is different. Patient beneficence and the avoidance of harsher forms of coercion by allowing some consentless measures are emphasised in empirical studies.^16,35–39^ Viewing a caring approach as a protection against the downsides of coercion has been previously described^2,16^ and aligns with nursing ideals.^40^ However, good nursing may also obscure ethical challenges. Jager and Perron discuss the ‘dance’ in which nurses’ skills, warmth, and flexibility render coercive elements invisible to both the patient and perhaps even the nurse.^41^ Indeed, participants struggled to make sense of the consentless measures they reported as common, especially since they do not resemble the ‘real coercion’ found in, for example, psychiatry.^16,31,42^

For the participants, consentless measures were connected with ward culture and efficiency. The way healthcare is organised has great potential to aid good ethics, but also risks hampering ethics and obscuring ethical concerns.^43–45^ The division of labour described by participants – whereby physicians make medical decisions at a distance from patients – places nurses, who work closer to patients, in an uncomfortable situation.^46^ The working environment described in this study risks undermining nurses’ professional autonomy^47,48^ and negatively impacting both patients and nurses.^43,49,50^ Ideally, the nurse serves as the patient’s advocate,^40^ a role that is especially important when interacting with vulnerable patients, as described in this study.^51,52^ It is particularly distressing that participants reported that older patients may be treated with less respect than younger patients. Furthermore, participants indicated that their loyalty to their patients was often in tension with their loyalty to the system. Similar findings have been described previously,^32^ which may explain why nurses accept consentless measures.^53^ While such divided loyalties might be expected to fuel moral distress,^53,54^ participants in this study only infrequently touched upon themes related to moral distress – for example, in the quotes involving ‘unacceptable coercion’.

Much previous research corroborates the finding that staff view physical coercion as worse than other methods of coercion.^2^ The finding that staff find ‘meaningless’ interventions particularly hard to justify is novel, although patients have been observed to share this attitude.^55^

When subjected to ethical scrutiny (all this study’s authors are also clinical ethicists), our contention is that staff place too much emphasis on providing healthcare regardless of consent, taking patient autonomy surprisingly lightly. Staff members’ self-reported difficulties in assessing coercion may lead them to downplay ethical challenges, and the paternalistic instinct to help patients who are perceived as unable to help themselves seems to be a strong driver. At the same time, nurses and other staff may, in part, be motivated by individual or group self-interest – for instance, desiring to be diligent workers, avoiding overly demanding workloads, or finding certain tasks tiresome. We further caution against the system-wide ageism mentioned by the participants, as well as the tendency to downplay many kinds of consentless measures because they are not interpreted as being ‘real coercion’. Any form of coercion must be justified by considerations of proportionality.^56^ If feasible alternatives exist or if the potential medical benefit is minimal, even mild consentless measures are ethically challenging.

The current study is one of few to examine coercive interventions in the somatic setting and raises intriguing questions meriting further research. It is imperative to examine the ideal of ‘not giving up’, which emerged as highly important in this study. Staff members’ reflections on meaningless versus meaningful care could also be further explored. Our study suggests that staff find it difficult to determine whether treatments are coercive, and vignette studies could be used to explore their understanding of voluntariness and coercion. In terms of clinical practice, our findings highlight the need for critical scrutiny of Swedish ward culture, more ethical reflection among staff, and improvements in inter-professional communication as well as nurses’ professional autonomy.

### Methodological considerations

Strengths of this study include its broad sampling from different internal medical wards at two differently sized hospitals, with the participant sample including nurses and other healthcare professionals. Sampling from wards with other patient categories may have yielded different results. Pilot testing of the interview guides revealed the possibility that the term ‘coercion’ distracted rather than assisted participants, leading to the choice of a broader term (consentless measures) and the use of examples to initiate discussions. A limitation of the study is the gender imbalance in its sample, which reflects the broader gender imbalance within nursing. To enhance analytical trustworthiness, triangulation was employed: JB is an MD and clinical ethicist, NJ is an academic and clinical ethicist, and TG is an RN and clinical ethicist, and each approached the material from their respective angles.

## Conclusions

This qualitative study indicates that nurses and other healthcare staff in Swedish medical wards have extensive experience with using, or witnessing colleagues use, consentless measures. A strong sense of duty to provide healthcare interventions may limit the consideration of patients’ consent. Participants often viewed the ‘consentless measures’ discussed as either easy to justify or requiring no justification at all. Justifications given included patients’ benefit, others’ benefit, or patients’ lack of decision-making capacity – findings consistent with previous studies in other contexts. However, further justifications related to how dissent is expressed, the reasons for a lack of decision-making capacity, and the necessity of ‘getting the job done’ have received less attention in the literature. Overall, participants seemed to take patient (non)-consent more lightly than what would be expected according to established norms in medical ethics.

## Supplemental Material

Supplemental Material - Ethical reflections of healthcare staff on ‘consentless measures’ in somatic care: A qualitative study Supplemental Material for Ethical reflections of healthcare staff on ‘consentless measures’ in somatic care: A qualitative study by Joar Björk, Niklas Juth, and Tove Godskesen in Journal of Nursing Ethics.

## Appendix

### Abbreviations

RN: registered nurse
AN: assistant nurse
MD: medical doctor
PT: physiotherapist
OT: occupational therapist
