# Supplementary material for: Ethical reflections of healthcare staff on ‘consentless measures’ in somatic care: A qualitative study
Source: Nurs Ethics. 2025 Apr 14;32(7):2227–40. doi: 10.1177/09697330251328649 (PMC12550209; doi:10.1177/09697330251328649)
Supplement: Supplemental Material - Ethical reflections of healthcare staff on ‘consentless measures’ in somatic care: A qualitative study [file sj-pdf-1-nej-10.1177_09697330251328649.pdf]

Research checklist
Consolidated criteria for reporting qualitative studies (COREQ): 32-item checklist

**Domain 1: Research team and reflexivity**		
Personal Characteristics		
1. Interviewer	JB	
2. Credentials.<br>What were the researcher's credentials?	JB: MD, PhD<br>NJ: PhD<br>TG: RN, PhD	
3. Occupation.<br>What was their occupation at the time of the study?	JB: MD, Researcher<br>NJ: Professor<br>TG: Professor	
4. Gender.<br>Was the researcher male or female?	JB: Male<br>NJ: Male<br>TG: Female	
5. Experience and training.<br>What experience or training did the researcher have?	JB: Considerable experience in qualitative research.<br>NJ: Considerable experience in qualitative research.<br>TG: Possess significant experience in conducting qualitative research across various research projects.	
Relationship with participants		
6. Relationship established<br>Was a relationship established prior to study commencement?	No relationship was established prior to study commenced.	
7. Participant knowledge of the interviewer<br>What did the participants know about the researcher?	One researcher (JB) had a prior superficial relationship with some participants before study commenced.	
8. Interviewer characteristics<br>What characteristics were reported about the interviewer/facilitator?	JB is interested in the subject of consentless measures. This interest is shared by the entire research group.	
**Domain 2: study design**		
Theoretical framework		Page
9. Methodological orientation and Theory<br><br>What methodological orientation was stated to underpin the study?	Reflexive Thematic Analysis.	Page 7
Participant selection		

10. Sampling<br>How were participants selected?	Purposive sampling.	Page 4
11. Method of approach<br>How were participants approached?	Focus groups interviews.	Page 5
12. Sample size<br>How many participants were in the study?	37	Page 4
13. Non-participation<br>How many people refused to participate or dropped out? Reasons?	Zero individuals declined participation	Page 5
14. Setting of data collection<br>Where was the data collected?	The focus groups were held in an undisturbed room at the medical department.	Page 6
15. Presence of non-participants<br>Was anyone else present besides the participants and researchers?	To the interviewer's knowledge no one else were present besides the participants and researchers.	
16. Description of sample<br>What are the important characteristics of the sample?	The participants are described in detail.	Page 5
Data collection		
17. Interview guide<br>Were questions, prompts, guides provided by the authors? Was it pilot tested?	Yes, semi structured.<br>The interview was pilot tested twice over. First by individual interviews with three RNs (separately), and then with one focus group (five RNs)	Page 5-6
18. Repeat interviews. Were repeat interviews carried out? If yes, how many?	No.	
19. Audio/visual recording. Did the research use audio or visual recording to collect the data?	Yes, we used an audio recorder.	Page 6
20. Field notes<br>Were field notes made during and/or after the interview or focus group?	No additional field notes were taken.	
21. Duration.<br>What was the duration of the interviews or focus group?	The interviews ranged from 53 to 81 minutes.	Page 6

22. Data saturation.<br>Was data saturation discussed?	Yes, the topic of data saturation was discussed (but we prefer the term “sufficient amount of information”)	Page 6
23. Transcripts returned.<br>Were transcripts returned to participants for comment and/or correction?	No.	
**Domain 3: analysis and findings**		
Data analysis		
24. Number of data coders.<br>How many data coders coded the data?	The first author conducted the initial coding of the data while the other authors read excerpts. All authors met and discussed the results until consensus was reached.	Page 7
25. Description of the coding tree. Did authors provide a description of the coding tree?	Table 3 displays the themes and subthemes identified in the analysis.	Page 8
26. Derivation of themes.<br>Were themes identified in advance or derived from the data?	The themes were not identified in advance but derived from the data.	Page 8
27. Software.<br>What software, if applicable, was used to manage the data?	Excel	Page 7
28. Participant checking.<br>Did participants provide feedback on the findings?	No.	
Reporting		
29. Quotations presented.<br>Were participant quotations presented to illustrate the themes / findings? Was each quotation identified?	Yes. All identifiers were removed from the transcribed interviews and pseudonymized with a code to ensure that identification of interviewees was not possible.	Page 8
30. Data and findings consistent.<br>Was there consistency between the data presented and the findings?	The authors have made an effort to keep the findings close to the original data and the interviewer recognized the discussions in the final results presentation.	Page 8-16
Were major themes clearly presented in the findings?	The major themes are described in the Results as well as in Table 3.	Page 8-16
32. Clarity of minor themes.<br>Is there a description of diverse cases or discussion of minor themes?	The subthemes are described in the text as well as in Table 3.	Page 8-16
